# N4-acetylcytidine-dependent GLMP mRNA stabilization by NAT10 promotes head and neck squamous cell carcinoma metastasis and remodels tumor microenvironment through MAPK/ERK signaling pathway

**DOI:** 10.1038/s41419-023-06245-6

**Published:** 2023-11-01

**Authors:** Yuanyuan Liu, Xing Wang, Yuying Liu, Jianqiang Yang, Wei Mao, Chen Feng, Xiaoliang Wu, Xinwei Chen, Lixiao Chen, Pin Dong

**Affiliations:** 1 Department of Otolaryngology: Head and Neck Surgery, Shanghai General Hospital, Shanghai Jiao Tong University School of Medicine, Shanghai, 200080 China; 2 Jiangxi Provincial People’s Hospital, The First Affiliated Hospital of Nanchang Medical College, Nanchang, 330046 China; 3 Centre for Medical Research and Translation, Jiangxi Provincial People’s Hospital, The First Affiliated Hospital of Nanchang Medical College, Nanchang, 330006 China; 4 Department of Hematology and Medical Oncology, Winship Cancer Institute, Emory University, School of Medicine, Atlanta, GA 30322 USA; 5 Department of Oncology, Shenzhen Hospital of Southern Medical University, Shenzhen, 510086 China

**Keywords:** Head and neck cancer, Head and neck cancer

## Abstract

N4-acetylcytidine (ac4C) is a post-transcriptional RNA modification that regulates in various important biological processes. However, its role in human cancer, especially lymph node metastasis, remains largely unknown. Here, we demonstrated N-Acetyltransferase 10 (NAT10), as the only known “writer” of ac4C mRNA modification, was highly expressed in head and neck squamous cell carcinoma (HNSCC) patients with lymph node metastasis. High NAT10 levels in the lymph nodes of patients with HNSCC patients are a predictor of poor overall survival. Moreover, we found that high expression of NAT10 was positively upregulated by Nuclear Respiratory Factor 1 (NRF1) transcription factor. Gain- and loss-of-function experiments displayed that NAT10 promoted cell metastasis in mice. Mechanistically, NAT10 induced ac4C modification of Glycosylated Lysosomal Membrane Protein (GLMP) and stabilized its mRNA, which triggered the activation of the MAPK/ERK signaling pathway. Finally, the NAT10-specific inhibitor, remodelin, could inhibit HNSCC tumorigenesis in a 4-Nitroquinoline 1-oxide (4NQO)-induced murine tumor model and remodel the tumor microenvironment, including angiogenesis, CD8^+^ T cells and Treg recruitment. These results demonstrate that NAT10 promotes lymph node metastasis in HNSCC via ac4C-dependent stabilization of the GLMP transcript, providing a potential epitranscriptomic-targeted therapeutic strategy for HNSCC.

## Introduction

Head and neck squamous cell carcinoma (HNSCC) is an aggressive disease that can develop in the oral cavity, pharynx and larynx. It is the sixth leading cause of cancer-related death, largely attributable to ineffective therapeutic options and the lack of early diagnostic markers with sensitivity and specificity [1]. Owing to its heterogeneity and complexity, the 5-year overall survival (OS) rate of HNSCC patients is still only about 50% [2]. Most patients with newly diagnosed HNSCC have regional lymph node (LN) metastases at onset [3]. Lymph node status is a key indicator of postoperative recurrence [4]. It has been revealed that the number of metastatic lymph nodes is negatively associated with the OS of patients with HNSCC after curative resection [5, 6]. Therefore, further elucidation of the molecular mechanisms underlying HNSCC LN metastasis is urgently needed to improve the prognosis of HNSCC patients.

Various factors can impact tumor initiation and metastasis, which is a dynamic and complex progressive process. Recently, mRNA modification has been reported to play an important role in tumor initiation and metastasis in different types of cancers [7–10]. There are numerous kinds of modifications on mRNA, including m6A, m5C, and ac4C, etc [11–13]. Although current studies mostly focus on m6A modifications in various diseases, other RNA modifications can also play critical roles in human disease, especially cancer progression. For example, the dysregulation of m5C modification results in the progression of bladder cancer [14]. Additionally, other new modifications remain largely unexplored when compared to m6A or m5C RNA modifications. Therefore, exploring new mRNA modification is necessary to understand the mechanisms of mRNA modification in cancer. In 2018, N4-acetylcytidine (ac4C) was first reported by the U.S National Cancer Institute, the only known “writer” protein of this mRNA modification is N-acetyltransferase (NAT10), which could maintain RNA stability, promote RNA translation, and increase the expression of ac4C modified mRNA [13]. As the only reported mRNA ac4C writer, NAT10 has been researched in many kinds of fields, including tumors and other multiple biological processes. For example, NAT10 was found to play an oncogenic role in bladder cancer through increasing BCL9L, SOX4, and AKT1 mRNA stability and translation efficiency depending on ac4C mRNA modification [15]. NAT10 was also identified to promote gastric cancer metastasis and EMT by ac4C modification on COL5A1 mRNA [16]. One study reported that NAT10 could accelerate PDAC cell lines growth in vitro [17]. A previous report showed that NAT10 could act as a potential biomarker for HNSCC [18], however, this study did not explore whether the mRNA ac4C modification could promote HNSCC progression and whether NAT10 has a role in LN metastasis of HNSCC or not. There are still few studies on the specific role and mechanism of NAT10-mediated mRNA acetylation modification in those researches. Therefore, further elucidation of the comprehensive molecular mechanisms of NAT10-mediated mRNA acetylation modification is of great significance for understanding the role of that new mRNA modification in tumor spread.

In this study, we set out to evaluate the positive correlation between increased expression of NAT10 with lymphatic metastasis and decreased survival of patients with HNSCC. Specifically, Nuclear Respiratory Factor 1 (NRF1) binded to the promoter of NAT10 and promoted its transcription. In vitro and in vivo functional assays with NAT10-knockdown or NAT10-rescued HNSCC cell lines showed strong NAT10 expression in lymphatic metastasis of HNSCC. Glycosylated lysosomal membrane protein (GLMP) was first described as a nuclear protein that regulates gene transcription. Recently, GLMP has been proved to be a lysosomal membrane protein associated with dysregulated lipid metabolism in NAFLD and NAFLD-associated HCC [19]. However, the roles of GLMP in HNSCC progression remain largely unknown. In our study, we elucidated the underlying mechanism by which NAT10 promotes cancer metastasis via GLMP mRNA ac4C modification, an important mechanism in stabilizing the target mRNA, to evoke oncogenic MAPK/ERK signaling. Remodelin, a specific small-molecule inhibitor of NAT10, remarkably inhibited the progression and remodeling the tumor microenvironment (TME) of HNSCC in 4-Nitroquinoline 1-oxide (4NQO)-induced murine OSCC tumors, suggesting that suppressing NAT10 may be a novel therapeutic manner for HNSCC patients.

## Methods

### Clinical samples and cell lines

In our study, 43 HNSCC tumor tissues and 20 corresponding normal tissues were obtained from patients undergoing surgical treatment at Shanghai General Hospital (Shanghai, China). Fresh tissues were well stored at −80 °C for further protein and RNA extraction. This study was approved by the Ethics Committee of the Shanghai General Hospital. Informed consent was obtained from all the patients prior to study participation.

### Cell lines and cell culture

The HN6 and FaDu cell lines were purchased from the Cell bank of the Chinese Academy of Sciences (Shanghai, China). They were incubated with 90% DMEM medium (Gibico, USA), 10% fetal bovine serum (Gibico, USA) and 100 U/ml penicillin (Gibico, USA) under a comfortable environment of 5% CO_2_ at 37 °C. We ensured that all cell lines were authenticated by STR profiling and tested free of mycoplasma infection.

### Construction of stable knockdown and overexpressed HNSCC cell lines

To construct stable NAT10- or GLMP-depleted HNSCC cell lines, short hairpin RNAs (shRNAs) specifically targeting NAT10 and GLMP were purchased from Sigma-Aldrich and cloned into the lentiviral pLKO.1-puromycin vector. To construct NAT10- or GLMP-overexpressing HNSCC cell lines, human NAT10 or GLMP-coding sequences were purchased from PPL (China) and inserted into the pLVC-IRES vector. The sequences of shRNA are shown in Table S1. Lentiviruses were packaged with PSPAX2 and PMD2.G. Briefly, 5 μg pMD2.G, 10 μg PSPAX2, 10 μg constructs for overexpression NAT10 and GLMP or knockdown of NAT10 and GLMP were co-transfected into HEK-293T cells in a 10 cm cell culture dish with PEI (26292-250, Polyscience). After that, the indicated lentiviruses were transfected into HNSCC cell lines and were selected using 2 μg/mL puromycin (ST551, Beyotime Biotechnology) for 10 days, with the knockdown and overexpression efficiency determined by RT-qPCR and western blot assay.

### Popliteal lymphatic metastasis model

Male 4–6-week-old nude mice were purchased from Cyagen (Shanghai, China) and used as the xenograft models. 5 × 10^6^ FaDu cells transfected with sh-NC-luc and sh-NAT10-luc were suspended in 60 µl PBS and injected into the footpads of the mice. Six weeks after injection, mice were imaged to assess lymphatic metastasis by injecting intraperitoneally with D-Luciferin sodium salt (200 μL, 150 μg/mL, 40901ES, Yeasen, China), and imaging with a bioluminescence system (NightOwl II LB983, Berthold Technologies, Germany). Subsequently, the mice were euthanized and the popliteal lymph nodes were removed for measurement and immunohistochemistry (IHC) analysis. All animal experiments were approved by the Animal Ethics Committee of Shanghai First People’s Hospital.

### Mouse models for HNSCC

For induction of HNSCC in mice, we selected 6-week-old male C57BL/6 mice fed with drinking water mixed with 50 μg/mL 4-NQO (N8141, Sigma-Aldrich, USA) for 16 weeks and then supplied normal drinking water for additional 8 weeks. To detect the inhibitory effect of the NAT10-specific inhibitor remodelin in vivo, 5 mg/kg remodelin (T5648-1G, MCE, USA) was injected once two days by intraperitoneally for 4 weeks.

### Quantitative real-time fluorescence quantitative PCR (qRT-PCR)

Total RNA was extracted from tissues and cell lines by using RNAiso Plus (No.9108, TaKara, Japan), as described in the instructions. PrimeScript Reverse Transcription Master Mix (RR036A, Takara) was used to synthesize cDNA by reverse transcription of RNA, according to the manufacturer’s protocol. qRT-PCR was performed on an Applied Biosystems 7500 (Foster City, California, USA) using the TB Green PCR Premium Ex Taq II kit (RR820A, Takara). The primers used in the article are listed in Supplementary Table S1. GAPDH was used as an internal reference and the 2^–ΔΔCt^ method was used to determine gene relative expression.

### Western blot

The cells and tissues were lysed using RIPA lysis buffer containing 1% protease inhibitors (EpiZyme, Shanghai, China). Then the extracted supernatant was added to the 5 × loading buffer and incubated in a metal bath at 100 °C for 10 min. The protein concentration was detected using a protein quantitative detection kit (Beyotime, China). The protein samples were separated by 7.5% or 10% SDS-PAGE (EpiZyme, China), transferred to PVDF membranes (Millipore, Germany), and blocked by 5% skimmed milk powder for 2 h. Subsequently, the membranes were incubated with primary and secondary antibodies, respectively. The details of the antibodies referred are listed in Supplementary Table S2. Finally, protein signals were visualized with an enhanced chemiluminescence kit and captured by A Tanon5200 Multiintelligent imaging system (Bio Tanon, China).

### Immunohistochemistry (IHC) and scoring analyses

The immunohistochemical experimental process is described in a previous article [20]. In shortly, Paraffin sections were dewaxed and blocked with hydrogen peroxide to repair their antigens. The sections were then blocked with serum, incubated with an antibody, and stained with hematoxylin. The sections were then rinsed, re-stained, and sealed for further evaluation.

Three independent superior pathologists blinded to the clinicopathological information performed scoring using a light microscope. The IHC score was obtained by multiplying the staining distribution score and the signaling intensity scores. The signal intensity scores were stratified into four classes in the HNSCC tissues as follows: 0, 1, 2, and 3, which were categorized as no, weak, moderate, and strong signals, respectively. The staining distribution scores were calculated following to the percentage of positive cells: scores of 0, 1, 2, 3, and 4 indicate 0–5%, 5–25%, 25–50%, 50–75%, and 75–100% stained cells, respectively. The median value was selected as the cut-off value.

### Wound healing assay

A certain concentration of the cell lines was pipetted into six-well plates to ensure full growth on the second day. Then straight lines were drawn using 10 uL pipettes and photographed for retention. After 24 h of culture in serum-free medium, images were captured again to clarify the changes in cell migration ability.

### Transwell assay

The cells in the logarithmic phase were suspended in a serum-free medium and planted in the upper chamber of the invasion chambers (BD Bioscience, USA). The lower chambers were cultured in a medium containing 10% serum for 24 h. Then, the chambers were removed, fixed in 70% methanol, and stained with crystal violet for 30 min, respectively. The stained cells were finally visualized and photographed with a microscope (Leica, Wetzlar, Germany).

### RIP-qRCR

RIP-qPCR was conducted using an RIP-kit (Bes5101, BersinBio, China). According to the manufacturer’s protocols, we performed RIP assays in the HN6 and FaDu cell lines to evaluate and validate NAT10 and GLMP mRNA. In short, magnetic beads pre-coated with 3ug of antibodies against NAT10 (Santa Cruze, USA) or mouse immunoglobulin G (Abcam, UK) were incubated with pre-frozen cell lysates for more than 16 h at 4 °C. Next, Proteinase K was used to digest the beads containing immunoprecipitated RNA-protein complexes, and RNA extraction was performed using the RNAiso Plus (Code No.9108, TaKara, Japan). The following steps were used to quantify the levels of GLMP mRNA using RT-qPCR.

### acRIP-qRCR, acRIP-seq and RNA-seq

The acRIP experimental process has been described in a previous article [17]. The Magna Merip m6A kit (Sigma-Aldrich, USA) was used according to its instruction by replacing the m6A antibody with the ac4C antibody (Abcam, UK). AcRIP analysis of NAT10 depleted and overexpressed cells was carried out. Briefly, total RNA was randomly digested into nucleotide chains of about 100–200 bp, and the ac4C antibody and magnetic bead complexes were incubated with the disrupted RNA. After that, RNA was extracted using RNAiso Plus and analyzed by RT-qPCR. We utilized the IgG group as a negative control and the input group as an endogenous control. The main primers used for the acRIP-qPCR are listed in Table S1. AcRIP-seq and RNA-seq were performed and analyzed by Guangzhou Epibiotek Co, Ltd.

### Luciferase reporter assay

We constructed two luciferase reporter plasmids via inserting the full-length 3’-UTR of GLMP and the mutant sequence, in which the C in the CAC ac4C motif sequence on the 3’-UTR of GLMP was replaced by G, into the pmirGLO vector. For luciferase assays, HN6 and FaDu cell lines were seeded in 24-well plates at a concentration of 1 × 10^5^ cells per well. Those luciferase reporter plasmids above were transfected with liposomes (Thermo Fisher Scientific). After 24 h, we collected and detected the intensity of luciferase via dual-luciferase, following the manufacturer’s instructions (Beyotime Biotechnology). The relative luciferase intensity was normalized to Renilla luciferase activity.

### RNA stability assay

HN6 and FaDu cells were treated with 2 μg/mL actinomycin D (Beyotime Biotechnology) for 0, 1, 3, and 6 h in 12-well plates. The half-life of the GLMP mRNA was measured by linear regression analysis. To quantify the relative levels of GLMP mRNA, total RNA was extracted and estimated by the method described above.

### Statistical analysis

All sample sizes were large enough to ensure proper statistical analyses. Analyses were performed using GraphPad Prime 8.0 and SPSS Statistics 28. Unpaired Student’s t-tests and chi-square tests were used to compare the significant differences between groups. The statistical significance of several groups was determined using analysis of variance. Cox proportional hazard regression was applied to examine the factors associated with death, and survival curves were obtained by the Kaplan-Meier method. Statistical significance was set at *P* < 0.05; *P < 0.05; **P < 0.01; ***P < 0.001, ****P < 0.0001, ns, non-significant. Kaplan-Meier plots with the log-rank test were used to analyze survival data.

## Results

### NAT10 is a novel high expression gene in HNSCC lymph nodes

**Fig. 1:**
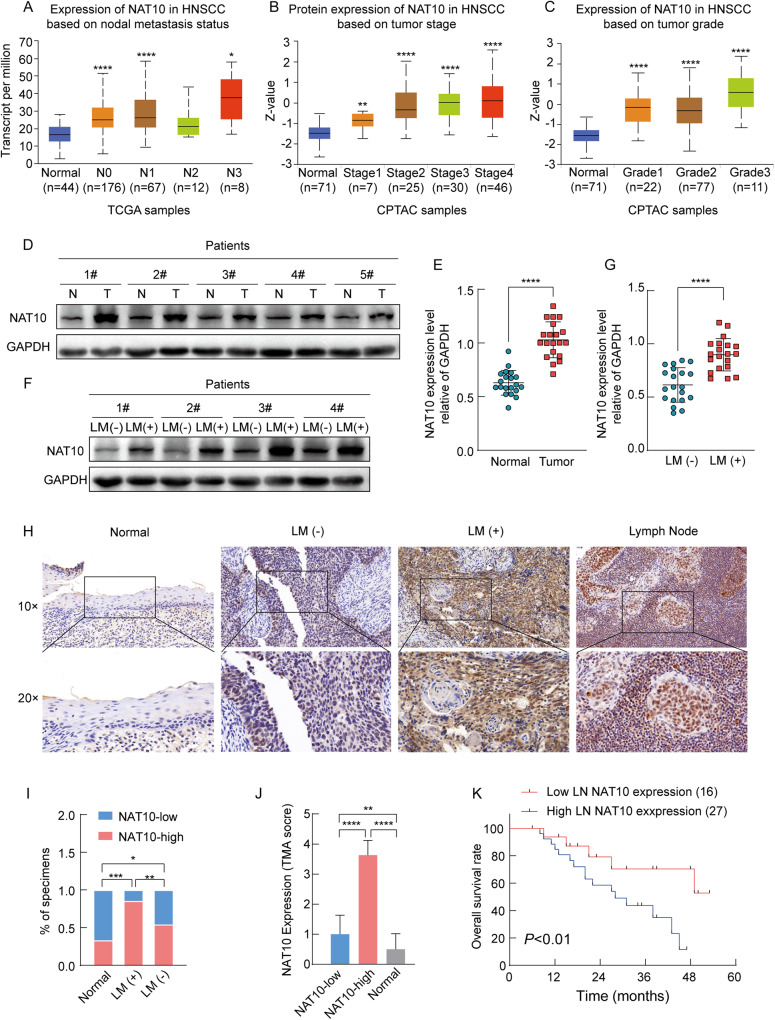
NAT10 highly expressed in HNSCC progression and associated with poor clinical outcomes in HNSCC patients. **A** Comparison of the mRNA expression level of NAT10 in HNSCC patients with different nodal metastasis statuses from the TCGA database. **B** Comparison of the proteogenomic expression of NAT10 in HNSCC patients with different stages from the CPTAC Data Portal. **C** Comparison of the proteogenomic expression of NAT10 in HNSCC patients with different grades from the CPTAC Data Portal. **D** Western blot analysis of NAT10 expression in adjacent normal (N) and tumor tissues (T) of five patients with HNSCC. **E** qRT-PCR analysis of NAT10 expression in adjacent normal (*n* = 20) and tumor tissues (*n* = 20) of patients with HNSCC. **F** Western blot analysis of NAT10 expression in four patients with HNSCC with or without lymphatic node metastasis. LM (−): non-lymphatic nodes; LM (+): lymphatic nodes. **G** qRT-PCR analysis of NAT10 expression in 20 patients with HNSCC with or without lymphatic node metastasis. **H** Representative images of NAT10 expression in normal, tumor tissues with or without lymphatic node metastasis, and lymph node metastasis. **I** Percentage of NAT10 expression in specimens of patients with HNSCC normal and tumor tissues with or without lymphatic node metastasis. **J** Bar chart of the distribution of NAT10 expression levels in normal, and tumor tissues with or without lymphatic node metastasis. **K** Overall survival (OS) analysis of 43 HNSCC patients with different NAT10 expression levels in lymphatic node metastasis. Data are shown as mean ± SEM. *P*-values were calculated by two-sided Student’s t-test in (**E**, **G**, **J**), two-sided Chi-Square test in (**I**) and two-sided log-rank test in (**K**). *P < 0.05; **P < 0.01; ***P < 0.001, ****P < 0.0001.

To determine the role of NAT10 in LN metastasis of HNSCC, we found that in TCGA database, HNSCC patients with advanced LNM showed significantly elevated NAT10 expression compared to patients with low LNM (Fig. 1A). Furthermore, in the Clinical Proteomic Tumor Analysis (CPTAC) data portal, as expected, the protein level of NAT10 was higher in patients with an advanced clinical stage than in those with low clinical stage (Fig. 1B). In addition, patients with high grade tumors exhibited significantly increased NAT10 protein levels compared to those with low grade tumors (Fig. 1C).

In our HNSCC patient specimens, qRT-PCR and western blot analyses demonstrated that NAT10 expression was higher in tumor tissues than in those of non-tumor tissues (Fig. 1D, E). Previous reports have shown that LN metastasis is significantly associated with poor prognosis of HNSCC patients [21]. Next, to explore whether NAT10 played a functional role in LN metastasis of HNSCC patients, resulting in a poor prognosis of HNSCC patient, we collected five pairs of fresh tissues of HNSCC patients with or without LN metastasis for evaluating NAT10 expression levels with qRT-PCR and western blotting, respectively. Our results showed that the mRNA and protein levels of NAT10 were increased in HNSCC patients with LN metastasis compared with patients without LN metastasis (Fig. 1F, G). To further investigate the clinical relevance of NAT10 in metastatic HNSCC, 43 cases of HNSCC primary tumor tissues and paired lymph node samples and adjacent non-tumor specimens were collected, and the expression levels of NAT10 were assessed. The results showed that the expression intensity of NAT10 was significantly higher in LNM-positive tumor tissues than in LNM-negative tumors and corresponding non-tumor specimens (Fig. 1H–J). Importantly, among patients with LN metastasis, high NAT10 expression was significantly related to poorer overall survival (Fig. 1K), indicating NAT10 could be a prognostic biomarker for patients with HNSCC.

### NAT10 promotes lymphatic metastasis in vivo

**Fig. 2:**
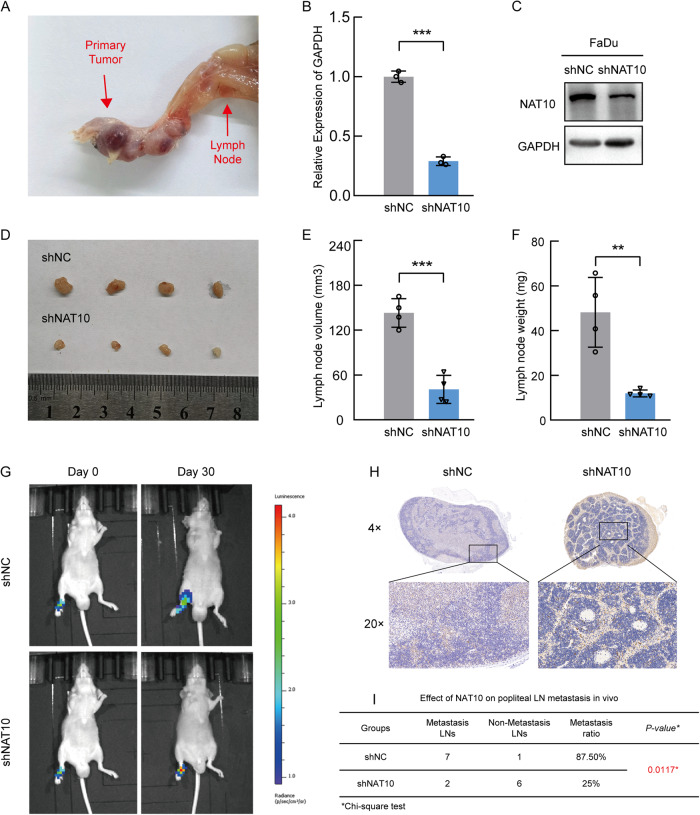
NAT10 promotes lymphatic node metastasis and lymphangiogenesis in vivo. **A** Representative images of the metastasized popliteal lymph node in nude mice. **B**, **C** qRT-PCR (**B**) and western blot (**C**) of analysis NAT10 expression in shNC and NAT10 deficiency in FaDu cells. **D** Images of popliteal LN metastasis of shNC and shNAT10 FaDu cell lines in the nude mice (*n* = 4). **E**, **F** The volume (**E**) and the weight (**F**) of popliteal lymph node for the shNC and shNAT10 group. **G** Representative bioluminescent images of popliteal LN metastasis of shNC and shNAT10 FaDu cell lines in the nude mice on days 0 and 30. **H** Representative images of lymph nodes invaded by FaDu cells and validated by CK7 IHC staining. **I** The metastatic ratio of popliteal LN calculated for the shNC and shNAT10 groups. Data are shown as mean ± SEM from three independently experiments. *P*-values were calculated by two-sided Student’s t-test in (**B**, **E**, **F**) and two-sided Chi-Square test in (**I**). **P < 0.01; ***P < 0.001.

To investigate the role of NAT10 in LN metastasis in HNSCC, we performed an in vivo assay with a popliteal LN metastasis nude mouse model, as previously described [4] (Fig. 2A). Briefly, we constructed a NAT10 knockdown and firefly luciferase FaDu cell line, and the efficiency of NAT10 deficiency was confirmed by qRT-PCR and western blotting (Fig. 2B, C). After that, these cells were injected into the footpads of nude mice. The effect of NAT10 on LN metastasis was measured by in vivo bioluminescence imaging, and the popliteal lymph nodes that represented the first and rate-limiting steps of cancer metastasis were examined after 4 weeks. Our results demonstrated that NAT10 depletion in FaDu cells resulted in fewer swollen and smaller popliteal lymph nodes (Fig. 2D–G) compared to the shNC control group. The infiltration of FaDu cells was then verified by CK7 staining (Fig. 2H, I). Overall, our results indicate that NAT10 may promote the spread of HNSCC by enhancing LN metastasis in HNSCC cells

### NAT10 expression is positively regulated by NRF1

**Fig. 3:**
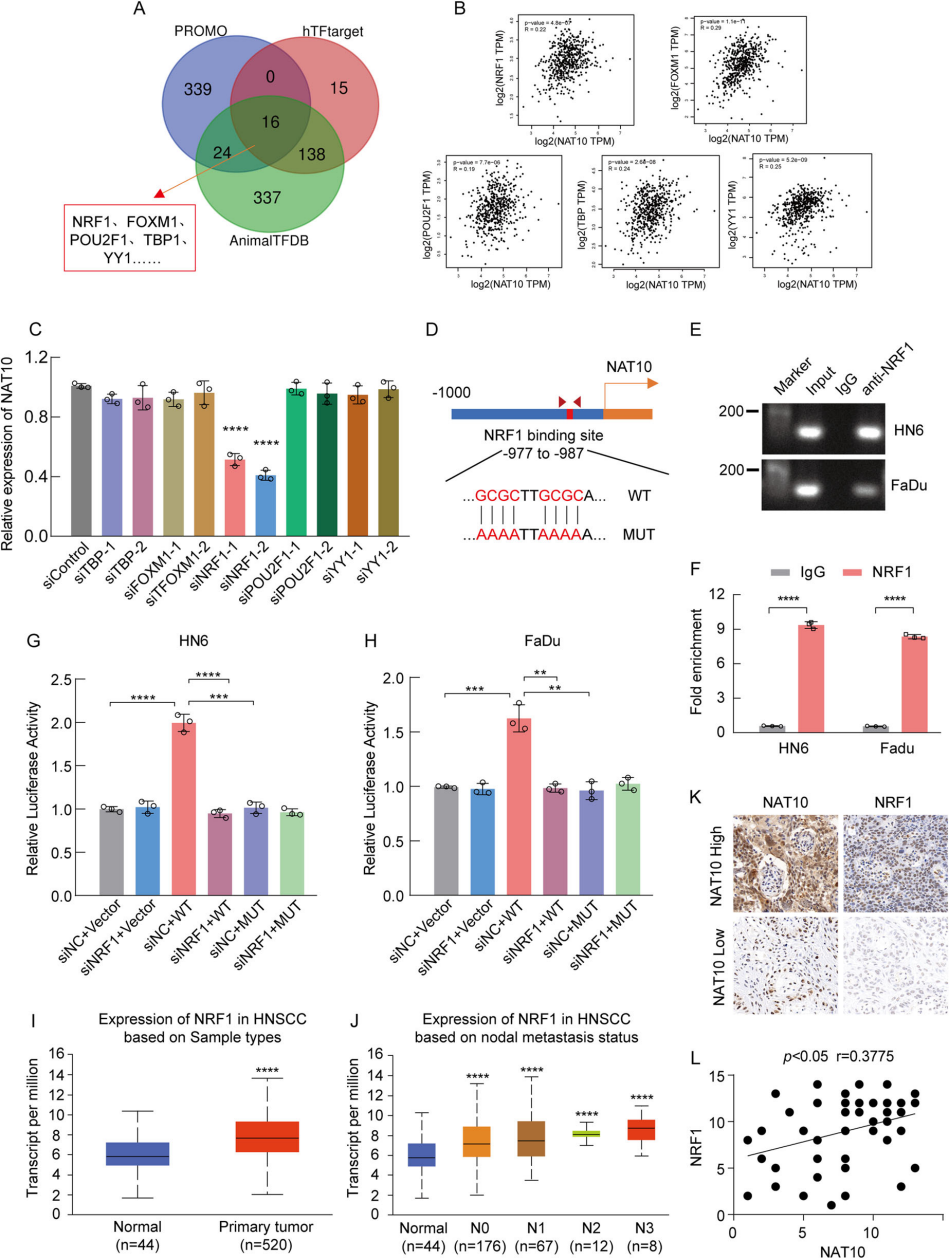
Transcription factor NRF1 increases NAT10 expression in HNSCC. **A** In silico analysis of possible TFs in NAT10 promoter region. **B** The correlation expression of NAT10 and potential TFs in TCGA-HNSCC cohorts in GEPIA2. **C** Relative NAT10 mRNA levels in HNSCC cells with or without depletion of each of the five TFs indicated in (**B**). **D**, **E** The putative NRF1 binding site in NAT10 promoter and the primers utilized for chromatin immunoprecipitation (ChIP) assays. **F** Images of agarose gel electrophoresis of the qPCR products. **G**, **H** Luciferase reporter assays in HNSCC cells co-transfected with the indicated vectors or siRNA for 48 h. **I**, **J** The expression levels of NRF1 in tumor and non-tumor tissues (**I**) and based on node metastasis status (**J**). **K** Representative images showing positive correlations between protein levels of NAT10 and NRF1 in HNSCC in specimens. Scale bar, 100 μM. **L** Spearman’s correlation analysis between protein levels of NAT10 and NRF1 in tumors. Data are shown as mean ± SEM at least three independent experiments. *P*-values were calculated by two-sided Student’s t-test. **P < 0.01; ***P < 0.001; ****P < 0.0001.

To explore the reason for the high overexpression of NAT10 mRNA in HNSCC, we performed an in silico analysis using three public website online tools (PROMO, AnimalTFDB and hTFtarget) to identify cis-elements in the NAT10 promoter region (from -1000bp to the transcription start site). Subsequently, 16 potential transcription factors (TFs) for NAT10 were found (Fig. 3A). Next, we evaluated the expression correlation between NAT10 and TFs in HNSCC using the GEPIA2 website (Fig. 3B). We identified five NAT10 TFs, of which only silencing NRF1 markedly decreased NAT10 mRNA levels in HNSCC cells (Fig. 3C). Next, we performed an in silico analysis showing that the cis-element of NRF1 may be located between -977 to -987bp upstream of the NAT10 transcriptional start site, which was confirmed by chromatin immunoprecipitation (ChIP) experiments (Fig. 3D–F). Furthermore, luciferase reporter experiments indicated an increase in the transcriptional activity of the NAT10 wild-type promoter compared to the control plasmid and the plasmid with the putative NRF1 binding motif mutant NAT10 promoter. This increase in luciferase intensity was reversed when NRF1 was knocked down (Fig. 3G, H). Meanwhile, the UALCAN website showed that in TCGA-HNSCC cohorts, the expression level of NRF1 was higher in tumor tissues than in non-tumor tissues, as well as in the nodal metastasis status (Fig. 3I, J). We further explored the correlation between NRF1 and NAT10 based on our clinical specimens and found a significant positive correlation (*p* < 0.05, *r* = 0.3775), especially in patients with lymphatic metastases (Fig. 3K, L). These results suggest NRF1-dependent positive regulation of NAT10 mRNA expression in HNSCC.

### Identification of NAT10-mediated N4-acetylation modification transcripts

**Fig. 4:**
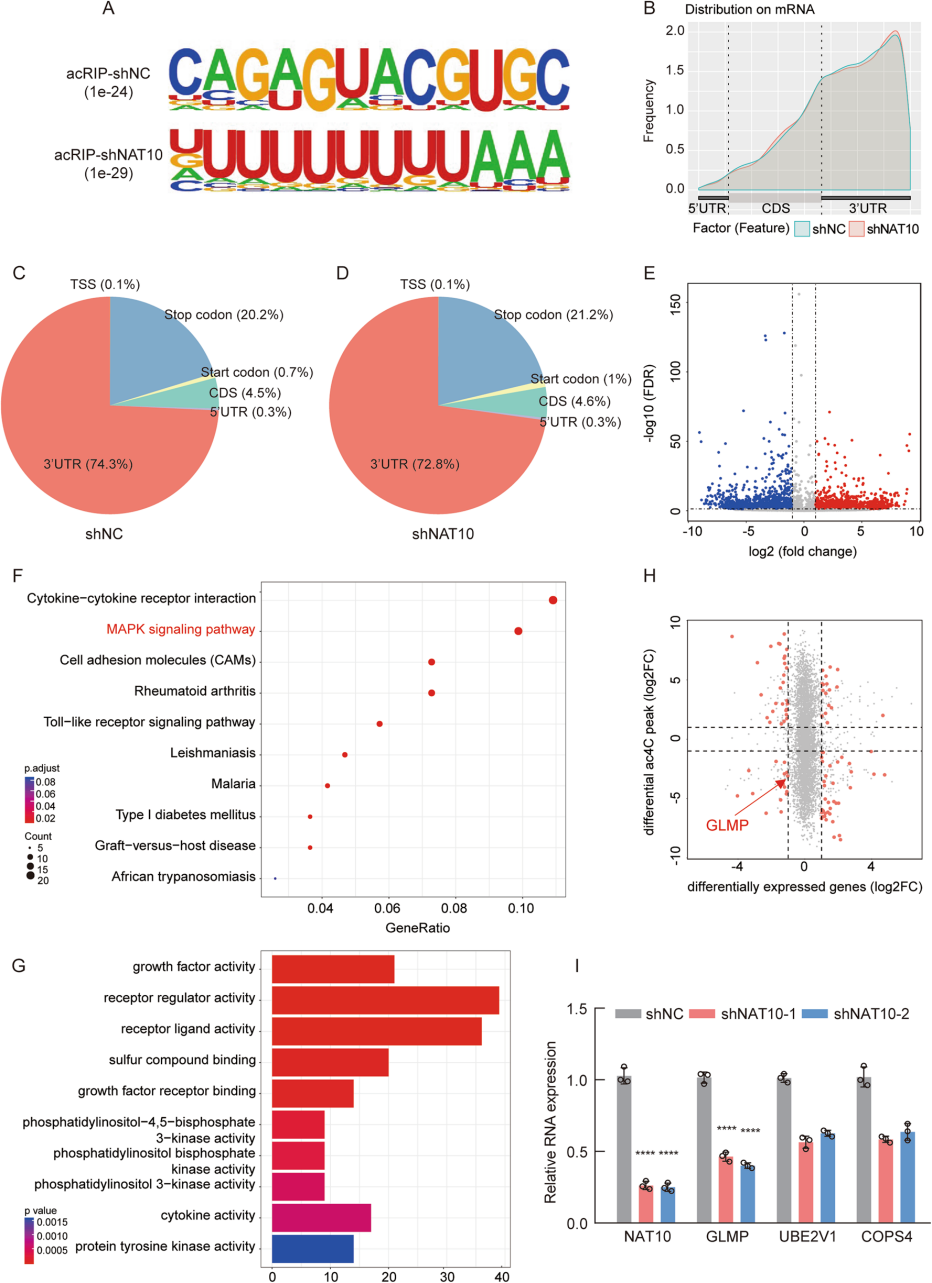
NAT10 modified ac4C mRNA regulates the progression of HNSCC cells. **A** The ac4C significant consensus motif of shNC and shNAT10-FaDu cells identified by HOMER. **B** Frequency of ac4C-containing peaks across the mRNA transcripts in both shNC and shNAT10-FaDu cells is shown in the metagene plot. **C**, **D** Pie chart of peak distribution showing the percentage of total ac4C peaks in the indicated regions including 5’-UTR, coding sequence, 3’-UTR and stop codon. **E** Volcano diagram showing the fold change of the differential expression of genes in shNC versus NAT10 knockdown in FaDu cells. **F** KEGG pathway enrichment analysis found the enriched pathway of down-regulated RNA levels after NAT10 deficiency. **G** GO analysis found the enriched pathway of down-regulated RNA levels after NAT10 deficiency. **H** Quadrant diagram for combined analysis of acRIP-seq and RNA-seq showed the levels of mRNA acetylation and transcription. **I** qRT-PCR assay showing the expression levels of the candidate genes in NAT10 silencing. Data are shown as mean ± SEM. *P*-values were calculated by two-sided Student’s t-test. ****P < 0.0001.

To assess the potential ac4C-regulated mRNA targets modified by NAT10 in HNSCC, acRIP-sequencing (acRIP-seq) assays were performed using mRNA isolated from shNC and shNAT10 FaDu cell lines. Based on the acetylated function of NAT10, a cytidine (C)-rich sequence motif was characterized as CXX; this motif was destroyed in the NAT10 deficiency group (Fig. 4A). We also found that ac4C modification distributed predominantly around the 3′-UTR and stop codons in both the shNC and shNAT10 groups (Fig. 4B–E). KEGG pathway enrichment and gene ontology (GO) analyses were also performed. KEGG pathway analysis showed that the ac4C modification-downregulated genes were significantly enriched in gene sets involved in cytokine-cytokine receptor interactions and the mitogen-activated protein kinase (MAPK) signaling pathway after NAT10 silencing (Fig. 4F). GO analysis demonstrated that the ac4C modification-downregulated genes were significantly enriched in gene sets and were significantly associated with growth factor and receptor regulator activities (Fig. 4G). These two biological results show that NAT10 may stimulate the growth factor signaling pathway to promote HNSCC progression.

After NAT10 silencing in FaDu cells, the ac4C and mRNA levels of the target genes decreased. Therefore, we combined the acRIP-seq and RNA-seq analyses to identify the targets whose ac4C modification and mRNA levels were decreased together in FaDu cells (Fig. 4H). To further verify our acRIP-seq results, we focused on three possible downstream target genes through qRT-PCR and found that the expression of GLMP was significantly downregulated in NAT10 knockdown FaDu cells (Fig. 4I). These results suggest that NAT10-mediated ac4C modification may be related to the overall mRNA abundance in FaDu cell line.

### GLMP is a downstream target gene of NAT10-mediated ac4C modification of mRNA

**Fig. 5:**
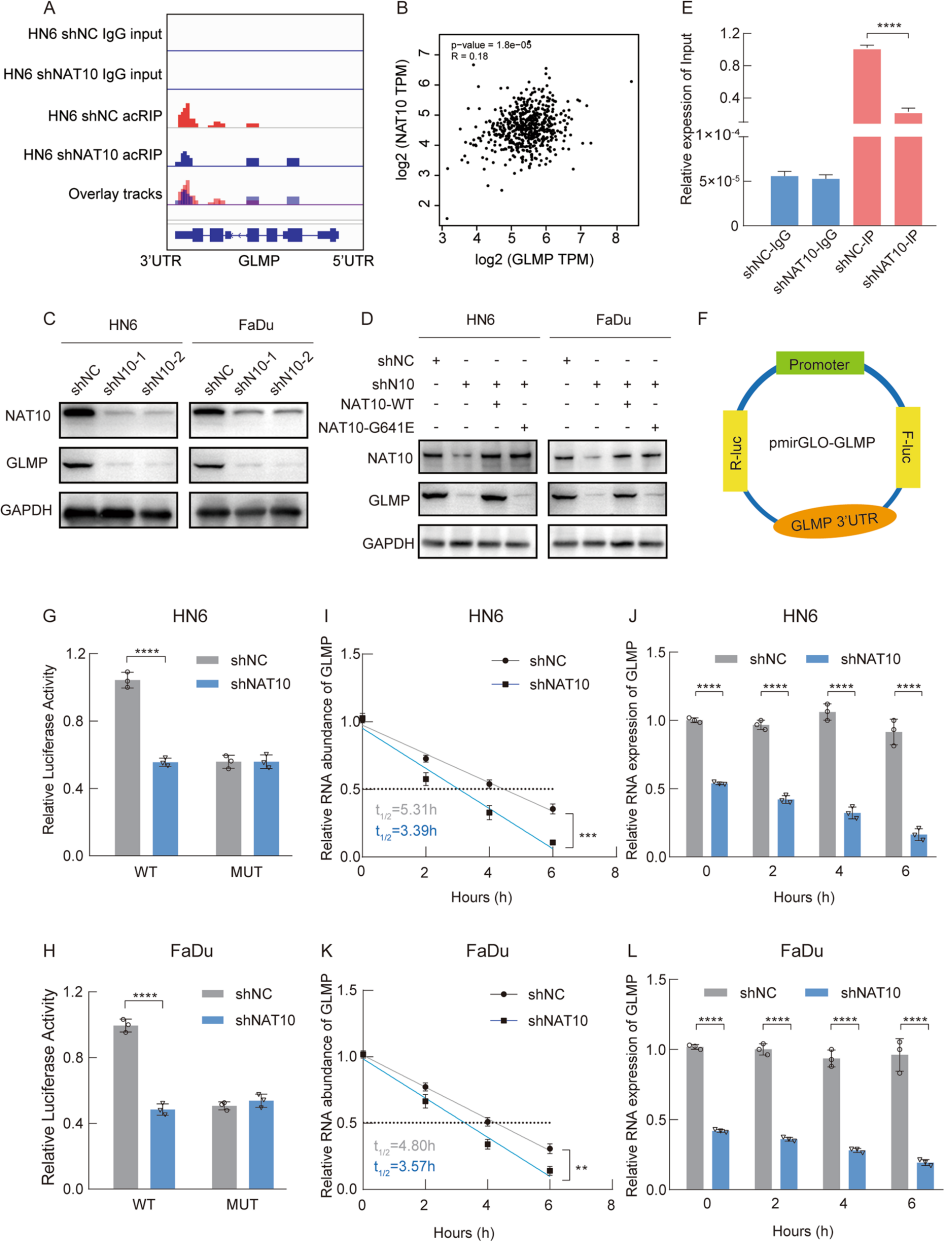
GLMP is a downstream target gene of NAT10. **A** IGV tracks showing read distribution across GLMP mRNA for acRIP-seq. **B** GEPIA2 website showing the relationship of NAT10 and GMLP. **C**, **D** Western blot of the expression levels of GLMP in NAT10 silencing (**C**) and rescued wild-type and mutant (G641E) in NAT10 deficiency of FaDu and HN6 cell lines (**D**). **E** Detection of the alteration of ac4C-modified GLMP mRNA with or without knockdown of NAT10 via RIP-qPCR. **F** Wild-type and mutant GLMP were inserted into pmirGLO-GLMP reporter vectors, respectively. **G**, **H** Luciferase reporter assays measured the luciferase activities of GLMP-CDS WT or GLMP-CDS Mut in FaDu and HN6 cells. **I**–**L** The impact of NAT10 on GLMP mRNA stability validated by the RNA decay experiment in HN6 and FaDu cell lines. Data are shown as mean ± SEM at least three independent experiments. *P*-values were calculated by two-sided Student’s t-test. **P < 0.01; ***P < 0.001; ****P < 0.0001.

We utilized the IGV software to investigate the ac4C modification level of GLMP and observed a decrease in ac4C modification, which was distributed near stop codons, indicating a potential role of ac4C modification in stabilizing GLMP mRNA (Fig. 5A). In the TCGA-HNSCC database, the mRNA expression level of NAT10 was significantly and positively associated with GLMP on the GEPIA2 website (Fig. 5B). Western blot analysis of in vitro HNSCC models demonstrated that NAT10 deficiency cell lines exhibited reduced GLMP expression at the protein level (Fig. 5C). We also rescued wild-type and mutant NAT10 (G641E) in NAT10 suppression FaDu cell lines, which showed that wild-type NAT10 could recuse GLMP expression, but mutant NAT10 could not (Fig. 5D). These data indicate that NAT10 promotes GLMP expression.

Previous reports have confirmed that regulating ac4C modification on the mRNA 3’-UTR contributes to target stabilization [22]. To further explore whether NAT10 modulated GLMP mRNA expression level through ac4C modification, we performed RIP-qPCR assay, which showed a decrease in the abundance of ac4C modification on GLMP mRNA in NAT10-knocked HNSCC cells (Fig. 5E). Furthermore, luciferase reporter assays were carried out to validate the importance of ac4C modification in the 3’-UTR of GLMP. We constructed both wild-type and mutant GLMP reporter genes (Fig. 5F). In the mutant form of GLMP, the ac4C motif, CAC, was replaced by GAG, thus deleting the ac4C modification motif. Luciferase intensity of the wild-type GLMP reporter decreased upon NAT10 deficiency, while NAT10 inhibition had no obvious impact on the expression of the mutant GLMP luciferase reporter (Fig. 5G, H). Subsequently, RNA decay assays were performed to assess the effects of NAT10-regulated mRNA ac4C modification on GLMP mRNA expression. The results showed that NAT10 deficiency decreased GLMP mRNA stability in HNSCC cell lines (Fig. 5I–L). In conclusion, NAT10 may regulate GLMP expression through mRNA ac4C modification.

### GLMP plays an oncogenic role in HNSCC progression

**Fig. 6:**
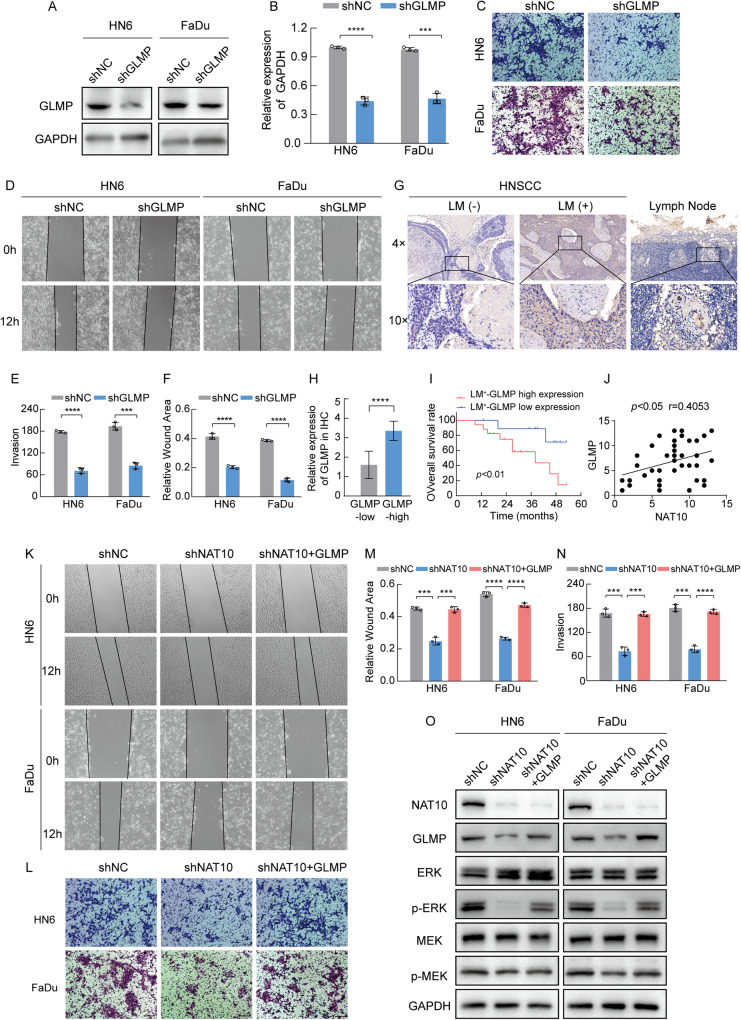
GLMP promotes HNSCC progression. **A**, **B** qRT-PCR (**A**) and western blot (**B**) of analysis GLMP expression in shNC and GLMP deficiency in HN6 and FaDu cells. **C**–**F** Wound healing assays of GLMP knockdown in HN6 and FaDu cells were recorded (**C**) and quantitatively analyzed (**E**). Images and quantification of transwell migration assays of GLMP knockdown in HN6 and FaDu cells were recorded (**D**) and quantitatively analyzed (**F**). **G** Representative images of GLMP expression in tumor tissues with or without lymphatic node metastasis, and lymph node metastasis. **H** Bar chart of the distribution of GLMP expression levels in HNSCC tumor tissues with lymphatic metastasis. **I** Overall survival analysis of 27 patients with HNSCC with different GLMP expression levels in lymphatic node metastasis. **J** Spearman’s correlation analysis between protein levels of NAT10 and GLMP in HNSCC tumor tissues. **K**–**N** Wound healing assays of rescuing GLMP in NAT10-depleted HN6 and FaDu cells were recorded (**K**) and quantitatively analyzed (**M**). Images and quantification of transwell migration assays of rescuing GLMP in NAT10-depleted HN6 and FaDu cells were recorded (**L**) and quantitatively analyzed (**N**). **O** Western blot analysis displaying that overexpression GLMP rescued the inhibitory impact of NAT10 knockdown on phosphorylation of MEK and ERK in HNSCC cells. Data are shown as mean ± SEM at least three independent experiments. *P*-values were calculated by two-sided Student’s t-test in (**B**, **E**–**H**, **M**, **N**) and two-sided log-rank test in (**I**). **P < 0.01; ***P < 0.001; ****P < 0.0001.

GLMP knockdown in HNSCC cell lines was performed to confirm the efficiency of knockdown and overexpression at both the mRNA and protein levels (Fig. 6A, B). These cell lines were used to evaluate the effects of GLMP on migration and invasion, which showed that GLMP knockdown suppressed migration and invasion (Fig. 6C–F). We further investigated whether GLMP promotes HNSCC progression. RNA-seq of TCGA-HNSCC data showed that GLMP expression was elevated in HNSCC tumor tissues (Fig. S1A). UALCAN portal analysis of GLMP expression in HNSCC based on nodal metastasis indicated that GLMP expression was significantly higher in HNSCC tissues with N0, N1, N2, and N3 LN metastases than in normal tissues, as well as in advanced tumor stages and grades (Fig. S1B–D). Kaplan-Meier analysis revealed that HNSCC patients with high GLMP mRNA levels had shorter overall survival times (OS) than those with low levels (Fig. S1E). To investigate the expression pattern of GLMP, we performed immunohistochemical staining to analyze its expression in HNSCC patient tissues and found that the expression levels of GLMP were significantly higher in LNM-positive HNSCC samples than in paired normal tissues and other LNM-negative tumor tissues (Fig. 6G, H). We conducted an OS analysis among 27 HNSCC patients with lymphatic metastasis and found that high GLMP expression was significantly correlated with poor overall survival (Fig. 6I). We further assessed the correlation between IHC staining scores and expression levels in our clinical samples and found a positive correlation between GLMP and NAT10 expression in HNSCC tissues (*p* < 0.05, *r* = 0.4053) (Fig. 6J). Therefore, GLMP is suggested to play an oncogenic role similar to that of NAT10 in HNSCC metastasis.

### GLMP is an intermediate medium of NAT10 modulates HNSCC metastasis

In the present study, we confirmed the oncogenic roles of NAT10 and GLMP in metastasis. Moreover, the role of the NAT10/GLMP regulatory axis in promoting HNSCC metastasis requires further validation. NAT10-depleted HNSCC cell lines showed rescued GLMP expression as verified by western blotting (Fig. 6O). We next explored whether GLMP could reverse the effects of NAT10 depletion on HNSCC cell migration and invasion. We constructed HNSCC cell lines with GLMP re-expression in NAT10 knockdown HNSCC cell lines. As expected, GLMP overexpression partially restored the inhibition of HNSCC cell migration and invasion by NAT10 knockdown (Fig. 6K–N). These results indicate that the NAT10/GLMP axis promotes HNSCC metastasis.

Previous studies have shown that the MAPK pathway is a major downstream pathway involved in cancer progression [23]. According to our acRIP-sequencing (acRIP-seq) assay, NAT10-mediated ac4C modification may promote HNSCC tumor progression by regulating the MAPK pathway (Fig. 4F). To explore whether NAT10 regulates the MAPK/ERK signaling pathway via GLMP, we performed rescue assays by overexpressing GLMP in NAT10-depleted cell lines and examined the levels of MEK, p-MEK, ERK, and p-ERK. The results showed that NAT10 inhibition decreased p-MEK and p-ERK levels, but not MEK and ERK levels, and rescued GLMP, which could partially restore the activation of the MAPK/ERK signaling pathway in NAT10-depleted cells, suggesting that GLMP is a pivotal downstream target of NAT10 that activates the MAPK/ERK signaling pathway (Fig. 6O).

### Remodelin inhibits growth and regulates the tumor microenvironment of HNSCC in 4-NQO-induced murine tumor model

**Fig. 7:**
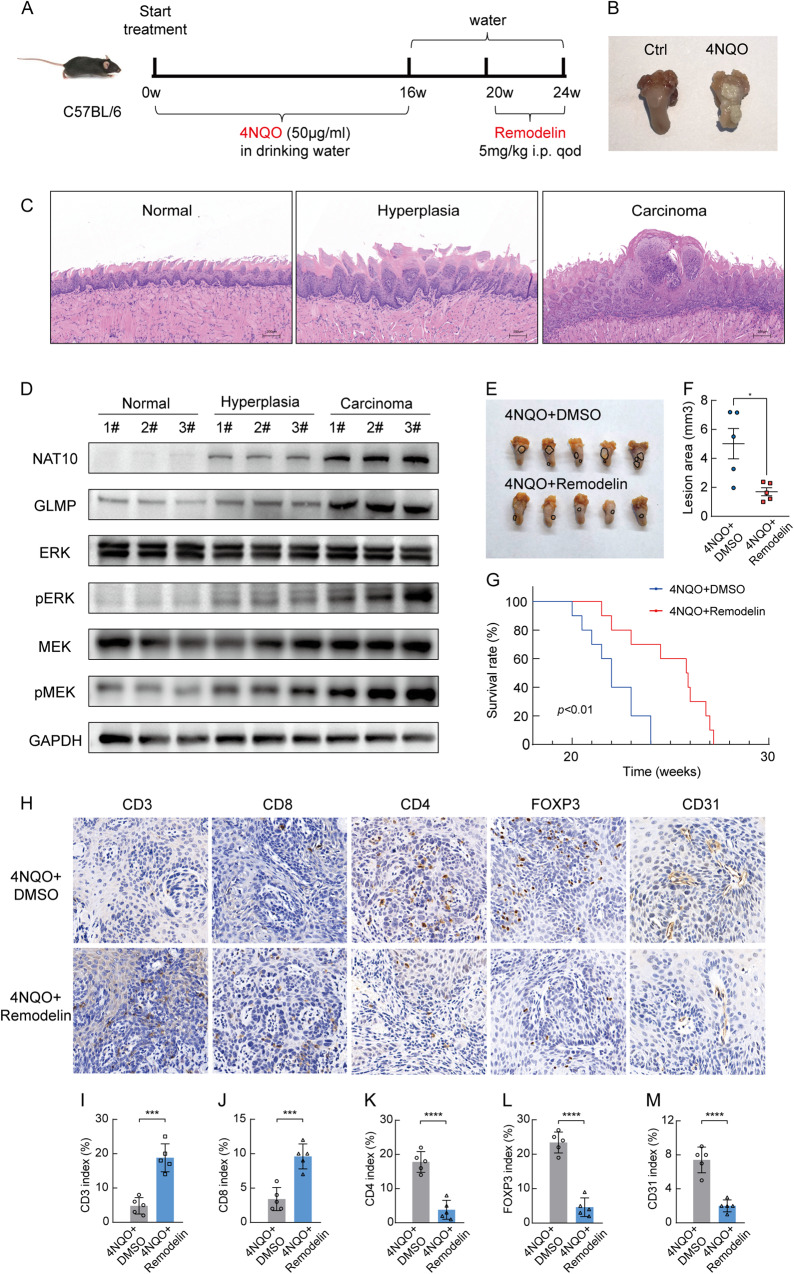
NAT10 exerts its oncogenic function, partially depending on GLMP in HNSCC. **A** Schematic diagram of the timeline of constructing the 4-NQO-induced OSCC mice model. **B** Representative images of OSCC induced by 4-NQO. **C** Representative pathological features of normal, hyperplasia and carcinoma induced by 4-NQO. **D** Western blot analysis of NAT10, GLMP, p-ERK and p-MEK expression levels in normal and different pathological stages from 4-NQO-induced OSCC mice models. **E**, **F** Images of tongue (**E**) lesions between non-treated and treated by remodelin and quantification of the tongue lesion area (**F**). **G** Overall survival of 4-NQO-induced OSCC mice model divided into the non-treated and treated by remodelin groups. **H**–**M** Representative of IHC (**H**) were displayed in randomly selected tumor and quantification of the relative intensities of IHC staining (**I**–**M**) was performed by image-pro plus 6.0 software. Data are shown as mean ± SEM at least three independent experiments. *P*-values were calculated by two-sided Student’s t-test in (**F**) and two-sided log-rank test in (**G**). *P* < 0.05 was considered significant; *P < 0.05; **P < 0.01; ***P < 0.001, ****P < 0.0001.

In our study, we explored the effects of NAT10 specific inhibitor-remodelin in a 4-NOQ-induced OSCC mouse model (Fig. 7A, B). Specimens of normal, tongue hyperplasia, and tongue carcinoma mice after 4-NQO withdrawal were obtained to detect the expression levels of NAT10 and GLMP using HE staining and western blotting. The results showed that NAT10 and GLMP expression levels increased in normal, hyperplastic, and carcinoma tissues (Fig. 7C, D). Next, we evaluated the efficiency of remodelin treatment and found that it significantly inhibited not only HNSCC tumorigenesis but also cell migration and invasion (Fig. 7E, F, S2). Meanwhile, remodelin treatment effectively prolonged the OS of these mice (Fig. 7G).

Moreover, RNA-seq results showed that NAT10 promoted the transcription of multiple critical cytokines such as CXCL3 and CXCL8 and suppressed CXCL10, CXCL11 and so on, which are important for the recruitment of immune cells, angiogenesis, tumor microenvironment remodeling, and cancer progression. As validated by qRT-PCR, NAT10 deficiency HNSCC cells showed decreased mRNA levels of CXCL3 and CXCL8 and increased mRNA levels of CXCL10 and CXCL11 (Fig. S3G–J).

**Fig. 8:**
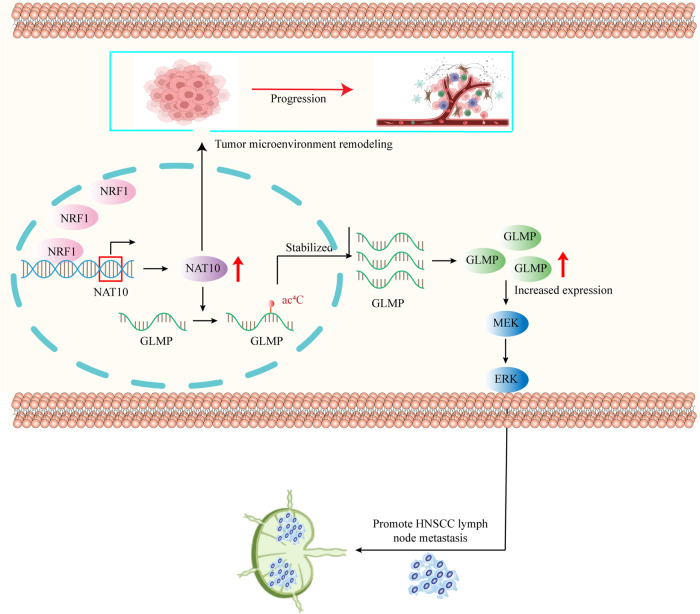
Graphical abstract describes how NRF1 upregulates NAT10 levels and NAT10 mediates LN metastasis and immune evasion in tumor cells.

Accumulating evidence has shown that the tumor microenvironment plays a pivotal role in HNSCC progression [24]. Therefore, we examined the status of tumor-infiltrating lymphocytes and angiogenesis. The results displayed that CD8^+^ T cells were elevated and Treg recruitment was inhibited in the remodelin-treated group, as indicated by CD3, CD8, CD4, and FOXP3. CD31 was used to evaluate angiogenesis and showed that the level of angiogenesis was downregulated in the remodelin-treated group (Fig. 7H–M). These results demonstrate that remodelin inhibits HNSCC tumorigenesis by increasing infiltrated CD8^+^ T cells and impairing angiogenesis and Treg recruitment in HNSCC tissues to reshape the tumor microenvironment, highlighting NAT10’s role as a potential therapeutic target (Fig. 8).

## Discussion

Tumor metastasis is a complex process involving multiple steps wherein cancer cells break away from their primary tumors, travel through the blood or lymph system, and build new tumors in distant organs. To escape from the initial site and form secondary tumors, cancer cells must first obtain the capabilities of migration and invasion, which are the key steps for subsequent infiltration into the circulation and the penetration into the capillary endothelium. In fact, tumor cells always settle in adjacent lymph nodes instead of going for a distant site immediately. Therefore, regional lymph node metastasis has been widely known as a criterion for determining cancer stage, which is closely related to the further spread of tumor cells in secondary organs.

LN metastasis is the primary cause of poor prognosis in patients with HNSCC [25]. Despite recent advances in therapeutic strategies, including surgery and targeted immunotherapy, treatment options for metastatic HNSCC are lacking [26, 27]. Thus, there is an urgent need to explore the potential molecular mechanisms of LN metastasis, and identify novel and efficient targets for the therapeutic approaches. In our study, we found that NAT10 is a potential prognostic marker for HNSCC. Moreover, NAT10 is required for lymphatic metastatic process, mainly because of its enhanced cell motility and invasiveness. In addition, NAT10 directly interacts with GLMP mRNA and promotes its mRNA stability, which is dependent on ac4C mRNA modification. These results provide new insights into the molecular mechanisms underlying LN metastasis.

NAT10 is a histone acetyltransferase that is primarily localized in the nucleolus and is associated with various biological processes [28–31]. NAT10 is the only reported enzyme that can catalyze ac4C modification in eukaryotic RNA [13]. Previous studies have also shown that ac4C acetylation regulates RNA synthesis, DNA damage repair, telomerase activity, and mRNA stability [32, 33]. It is also involved in the development, progression and prognosis of many human diseases, including inflammation and cancer [34–37]. NAT10 is always overexpressed in various cancers and is significantly associated with clinical features and poor prognosis [38–40]. To our knowledge, our findings are the first to show that higher NAT10 expression is obviously associated with LN metastasis and poor OS, demonstrating that NAT10 may act as a prognostic biomarker in HNSCC and function as an oncogenic factor in the lymphatic metastasis of HNSCC.

M6A mRNA modification has been validated to be correlated with lymphatic metastasis in different kinds of cancers [41–43]. For example, METTL3, an m6A writer, is significantly associated with LN metastasis and a poor prognosis in cervical cancer [42]. However, the role of NAT10 in LN metastasis of HNSCC remains unclear. In our study, we found that patients with lymphatic metastasis had higher NAT10 expression levels than those without lymphatic metastasis, and we speculated that NAT10 may promote lymphatic metastasis in HNSCC. To validate this, we used a popliteal LN metastasis model by injecting FaDu cells into the footpads of nude mice, which was a sensitive and quantitative method for evaluating lymphatic metastasis in vivo [21, 44]. Our results showed that NAT10 deficiency obviously suppressed lymphatic metastasis in vivo. Overall, our results revealed that NAT10 accelerated HNSCC progression by promoting lymphatic metastasis of HNSCC, demonstrating the broad clinical importance of NAT10 in cancer metastasis.

Another interesting finding was the identification of NRF1 as an NAT10 transcriptional activator. Through an integrated analysis, we found that NRF1 was a potential modulator of NAT10. NRF1 is a famous TF that has been proved to be overexpressed in HNSCC [45]. In this study, we showed that NRF1 was positively associated with NAT10 expression, supporting the hypothesis that NRF1 upregulates NAT10 expression. Furthermore, we indicated that NRF1 could bind to the promoter region of NAT10, increasing NAT10 transcription level. Overall, we showed for the first time that NAT10 overexpression in HNSCC may be partially regulated by cis-elements.

To further address the oncogenic role of NAT10-mediated ac4C modification in HNSCC, we performed acRIP and RNA-sequencing in HNSCC. Notably, genes with significant different expression levels were mainly enriched in the MAPK/ERK pathway. It is known that MAPK/ERK pathway plays an important role in various cellular processes [23, 46]. Dysregulation of the MAPK/ERK pathway is associated with many diseases including malignant tumors [47, 48]. However, the mechanism by which the MAPK/ERK pathway is over-activated in HNSCC remains largely unknown. In this study, we revealed that NAT10 overexpression induced over-activation of the MAPK/ERK signaling pathway by stabilizing GLMP mRNA via the modification of ac4C on GLMP mRNA. RNA stability assays indicated that the expression level of NAT10 was positively associated with GLMP mRNA stabilization. Overall, NAT10 was thought to stabilize GLMP mRNA rather than the translation efficiency by increasing the modification of ac4C in GLMP mRNA. Next, we explored whether NAT10 triggered the activation of the MAPK/ERK pathway depending on GLMP protein. By rescuing GLMP expression in NAT10-depleted cell lines, we found that NAT10 partially activated MAPK/ERK signaling by GLMP.

GLMP is known as a potential lipid regulator [19, 49]. Our study indicated that GLMP is the downstream target of the NAT10-regulated mRNA ac4C modification, which mediates HNSCC LN metastasis. GLMP causes chronic liver damage that progresses to liver fibrosis [50, 51]. However, the role of GLMP in cancer remains still largely unknown. In this study, GLMP played a vital role in LN metastasis of HNSCC through the MAPK/ERK signaling pathway. However, our study did not explore the molecular mechanisms underlying the promotion of HNSCC development by GLMP.

Finally, we evaluated the therapeutic value of the NAT10 specific inhibitor-remodelin in 4NQO-induced murine OSCC tumors through tumorigenesis and found that remodelin could inhibit tumor progression. Our further studies firstly found that remodelin increased the infiltrated CD8^+^ T cells, and suppressed the number of Treg and angiogenesis recruitment, which suggested that remodelin may be combined with immunotherapy to inhibit HNSCC progression.

Meanwhile, we recognize some limitations of this study. First, we showed that GLMP is the main target of NAT10-mediated ac4C modification in the promotion of HNSCC, further detection of some other functional targets is required. Second, the number of mice used in the remodelin treatment assay was small; thus, a larger sample size is needed before further clinical translation. Moreover, an in vivo assay of LN metastasis was performed using NAT10 knockdown HNSCC cells in nude mice, which should be complemented with other mouse models with a normal immunological background.

In conclusion, this study revealed that NAT10 plays a crucial role in the development of LN metastasis in HNSCC by modulating mRNA ac4C modification. With acRIP and RNA sequencing, we identified GLMP as the target gene of NAT10-mediated ac4C modification in HNSCC, which triggers the MAPK/ERK pathway. However, the possibility that several other genes promote LN metastasis in HNSCC by modulating other genes cannot be excluded. Our study also used NAT10 specific inhibitor-remodelin to treat an HNSCC mice model by 4-NQO, indicating that NAT10 may be a potential target for the treatment of HNSSC in the future. Collectively, our study not only indicates that NAT10 has an oncogenic role in LN metastasis of HNSCC and remodeling of the tumor microenvironment, but also suggests it as a potential therapeutic target for the novel treatment of HNSCC.

### Supplementary information

Supplementary figures and tables.

Original Data File

Reproducibility Checklist

## Data Availability

The original data of the article can be obtained from the corresponding author upon reasonable request. The acRIP-seq and RNA-seq raw data have been deposited in NCBI’s GEO under accession number GSE239900.
