# Supplementary material for: N4-acetylcytidine-dependent GLMP mRNA stabilization by NAT10 promotes head and neck squamous cell carcinoma metastasis and remodels tumor microenvironment through MAPK/ERK signaling pathway
Source: Cell Death Dis. 2023 Nov 1;14(11):712. doi: 10.1038/s41419-023-06245-6 (PMC10620198; doi:10.1038/s41419-023-06245-6)
Supplement: Supplementary file 1 — Supplementary figures and tables. [file 41419_2023_6245_MOESM1_ESM.pdf]

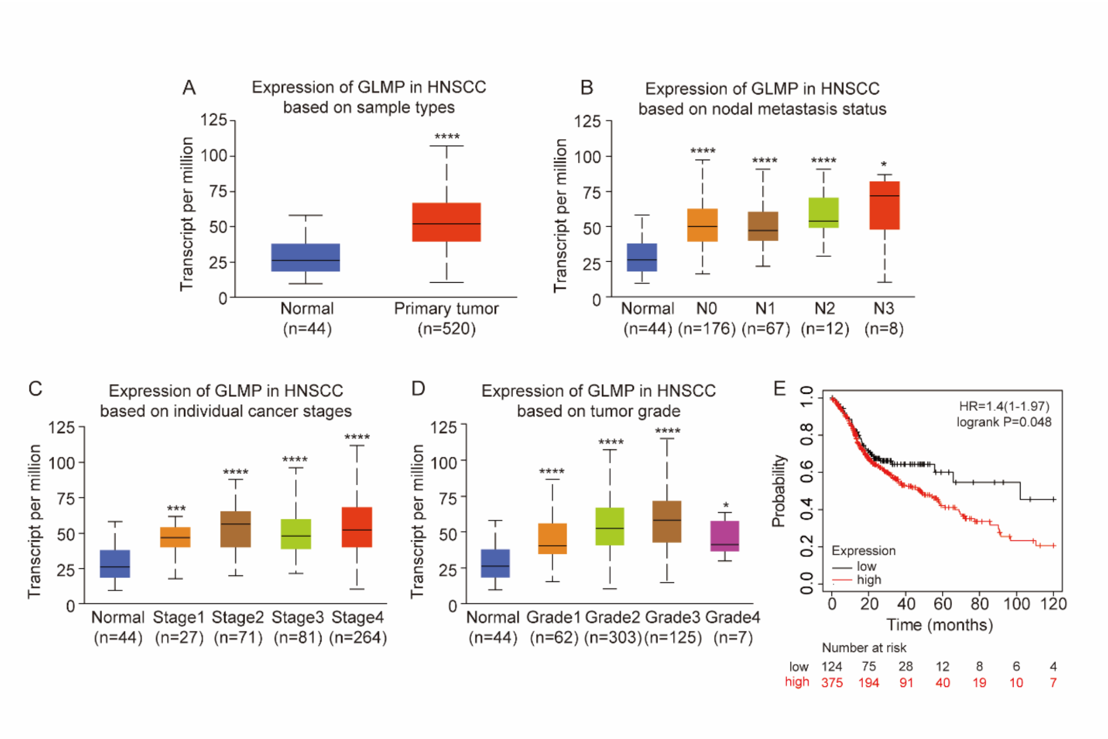

Figure S1. GLMP is highly expressed in HNSCC progression and associated with poor clinical outcomes in HNSCC patients. A Comparison of the expression level of GLMP in tumor and non-tumor specimens from UALCAN online tool. **B** Comparison of the expression level of GLMP in HNSCC patients with nodal metastasis status (N0-N3) from the UALCAN online tool. **C** Comparison of the expression level of GLMP in HNSCC patients based on cancer stages from the UALCAN online tool. **D** Comparison of the expression level of GLMP in HNSCC patients based on tumor grade from UALCAN online tool. **E** Overall survival of HNSCC patients with low and high GLMP levels from the Kaplan-Meier analysis. $P < 0.05$ was considered significant; *, $P < 0.05$; **, $P < 0.01$; ***, $P < 0.001$, ****, $P < 0.0001$.

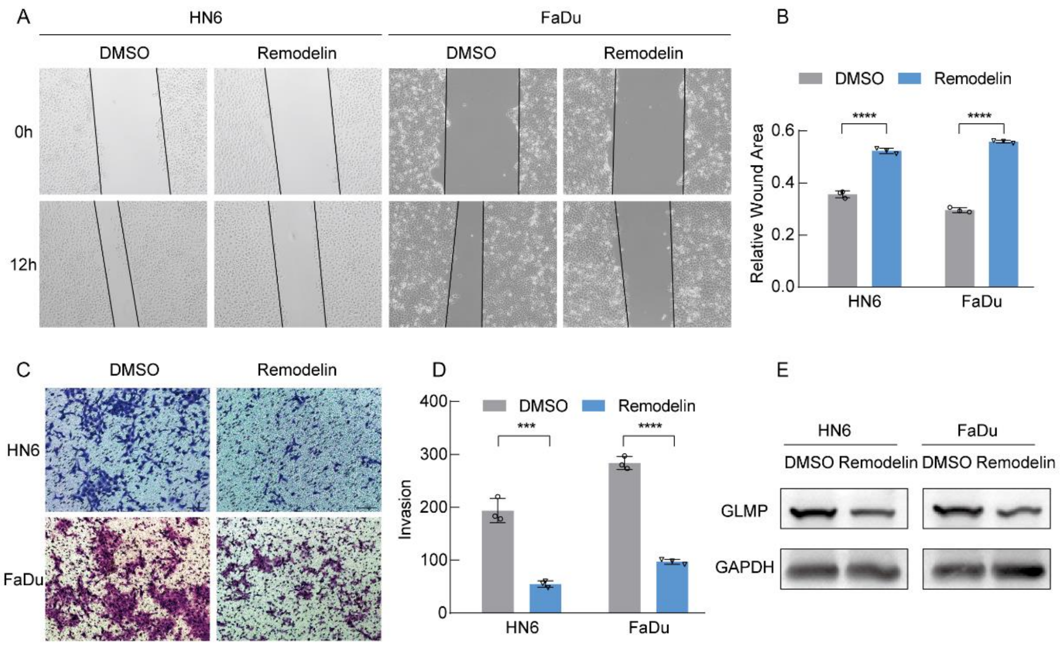

Figure S2 Remodelin inhibits HNSCC cells migration and invasion. A-B Wound healing assays of HN6 and FaDu cells with or without remodelin treatment were recorded (**A**) and quantitatively analyzed (**B**). **C-D** Transwell invasion assays of HN6 and FaDu cells with or without remodelin treatment were recorded (**C**) and quantitatively analyzed (**D**). The number of invasion cells was counted from three different fields. **E** Immunoblotting detected the protein levels of NAT10 in HN6 and FaDu cells with or without remodelin treatment. Data are shown as mean±SEM at least three independent experiments. *P* values were calculated by two-sided Student's t test. ***, $P < 0.001$; ****, $P < 0.0001$.

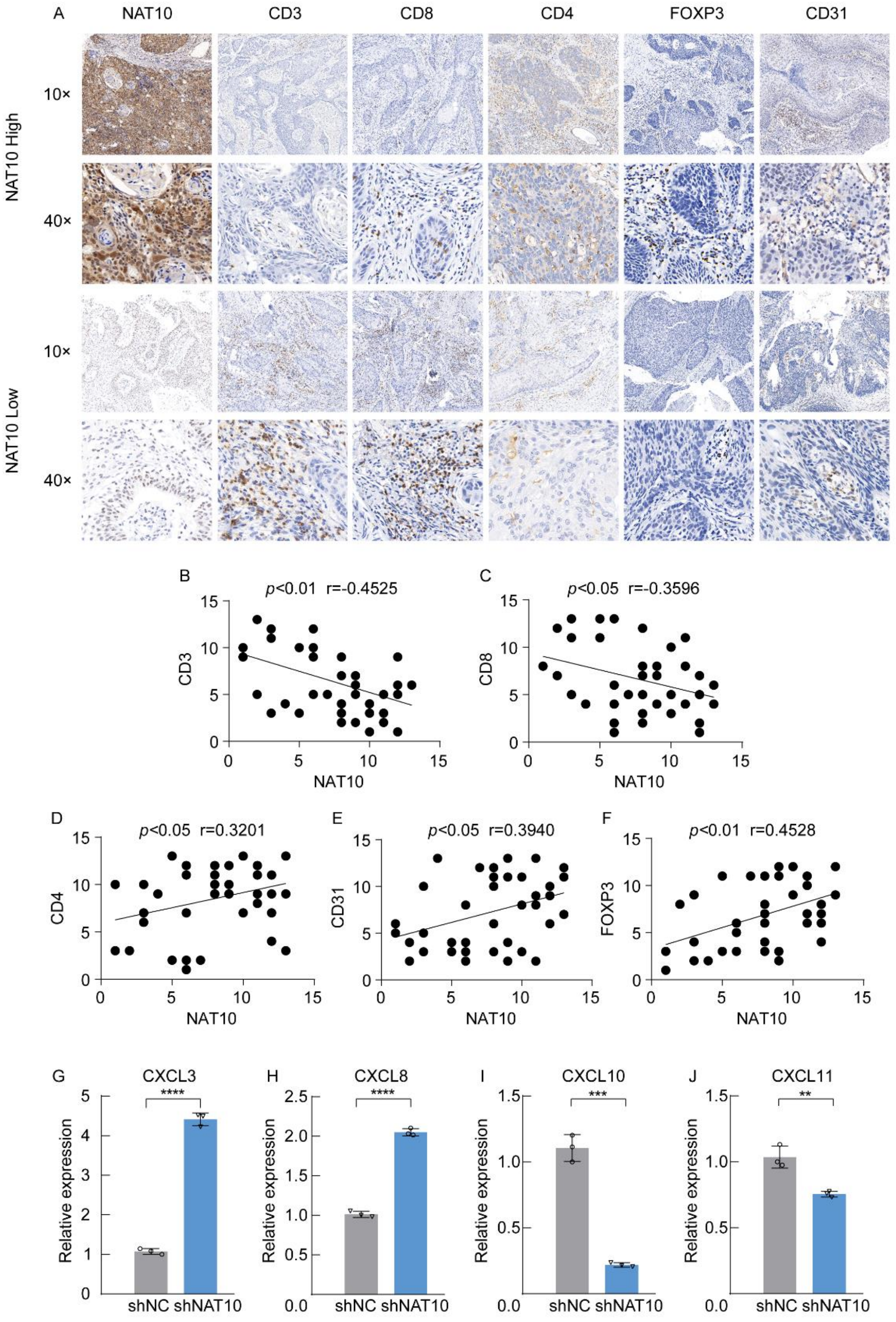

Figure S3 NAT10 remodels the tumor microenvironment in patient specimens. A Representative images showing correlations between protein levels of NAT10 and CD3, CD8, CD4, CD31, FOXP3 in specimens. Scale bar, 100uM. **B-F**. Spearman's correlation analysis between protein levels of NAT10 and CD3, CD8, CD4, CD31,

FOXP3 in specimens. **G-J** qRT-PCR of analysis showing cytokines expression in shNC and GLMP deficiency in FaDu cells. Data are shown as mean±SEM. *P* values were calculated by two-sided Student's t test. **, $P < 0.01$; ***, $P < 0.001$; ****, $P < 0.0001$.

Table S1: shRNA and main primer sequences

Oligonucleotides		
shNAT10-1:CGAGCTGGATTTGTTCCTGTT	Sangon Biotech	N/A
shNAT10-2:GCAATTGTACACAGTGACTAT	Sangon Biotech	N/A
shGLMP:TGGAGGTTGGAGCATCAAGTT	Sangon Biotech	N/A
NAT10-F: ATAGCAGCCACAAACATTCGC	Sangon Biotech	N/A
NAT10-R: ACACACATGCCGAAGGTATTG	Sangon Biotech	N/A
GLMP-F: AGCCTTTGGGAAGACCATATCC	Sangon Biotech	N/A
GLMP-R: TGGCCTTGAAATGTGGCACT	Sangon Biotech	N/A
GADPH-F: GGAGCGAGATCCCTCCAAAAT	Sangon Biotech	N/A
GADPH-R:GGCTGTTGTCATACTTCTCATGG	Sangon Biotech	N/A

Table S2: List of primary antibodies and their sources

Antibodies	SOURCE	IDENTIFIER
Mouse anti-NAT10	Santa Cruz Biotechnology	Sc-271770
Rabbit anti-GLMP	Abcam	Ab231317
Rabbit anti-CD3	Proteintech	GB13104
Rabbit anti-CD4	Servicebio	GB13064
Rabbit anti-CD8	Servicebio	GB114196
Rabbit anti-CD31	Servicebio	GB113151
Rabbit anti-F4/80	Servicebio	GB11027
Rabbit anti-CK7	Servicebio	GB13225
Rabbit anti-β-actin	Abclonal	AC026
Rabbit anti-NRF1	Abclonal	A3252
Rabbit anti-MEK1/2	Abcam	ab178876
Rabbit anti-phospho MEK1/2	Abcam	ab194754
Rabbit anti-ERK1/2	Abcam	ab17942
Rabbit anti-phospho ERK1/2	Abcam	ab76299
Rabbit anti-N4-acetylcytidine	Abcam	ab252215
