# Supplementary material for: N4-acetylcytidine-dependent GLMP mRNA stabilization by NAT10 promotes head and neck squamous cell carcinoma metastasis and remodels tumor microenvironment through MAPK/ERK signaling pathway
Source: Cell Death Dis. 2023 Nov 1;14(11):712. doi: 10.1038/s41419-023-06245-6 (PMC10620198; doi:10.1038/s41419-023-06245-6)

Fig1-D-NAT10

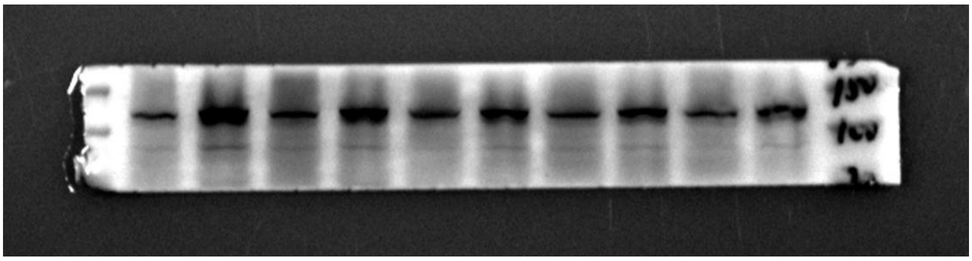

Fig1-D-GAPDH

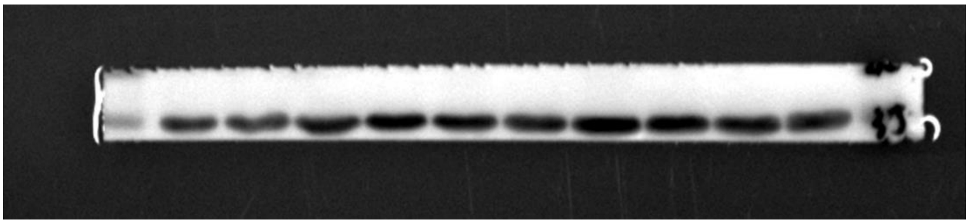

Fig1-F-NAT10

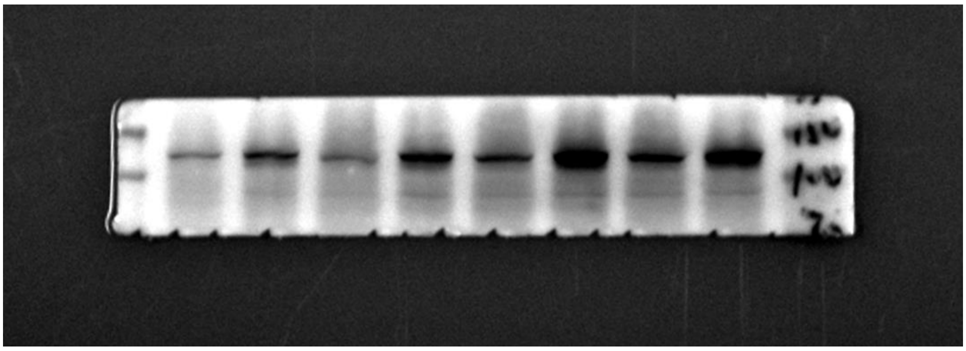

Fig1-F-GAPDH

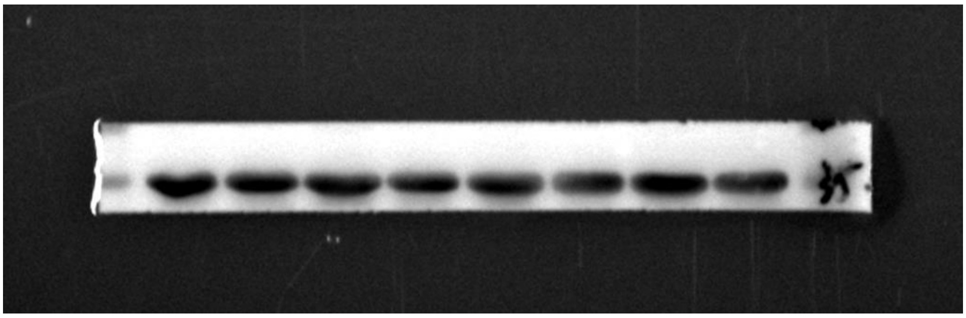

Fig2-C-NAT10

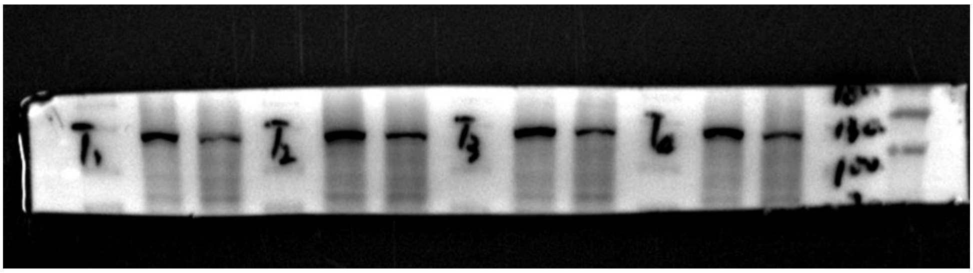

Fig2-C-GAPDH

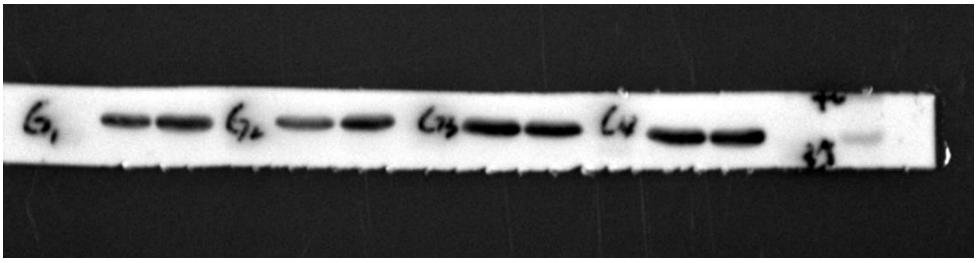

Fig5-C-NAT10

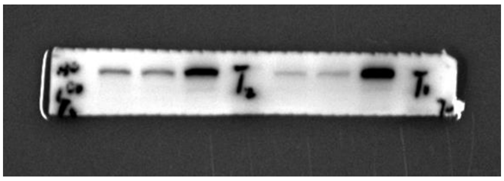

Fig5-C-GLMP

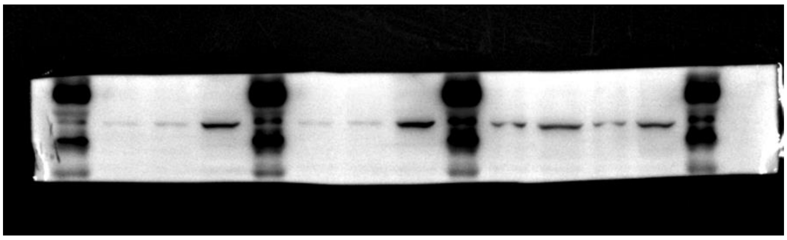

Fig5-C-GAPDH

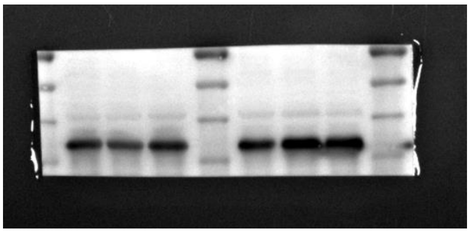

Fig5-D-NAT10

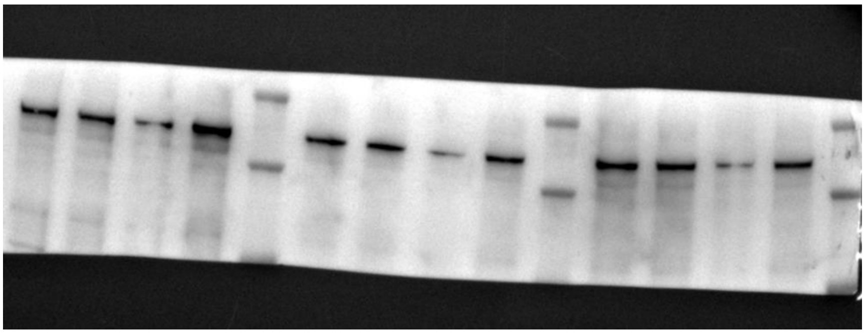

Fig5-D-GLMP

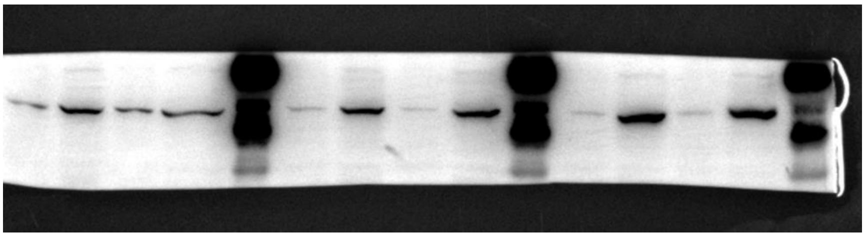

Fig5-D-GAPDH

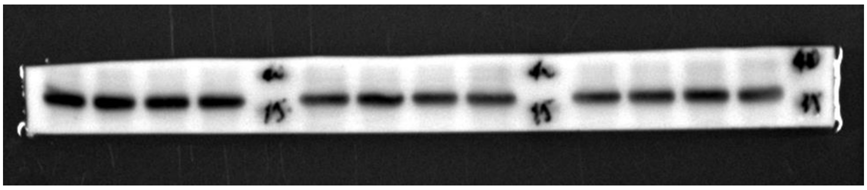

Fig6-A-GLMP

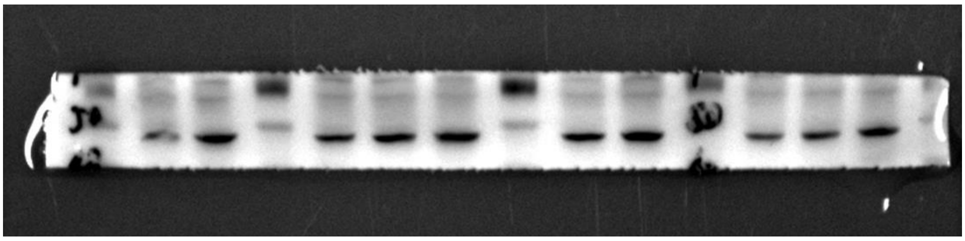

Fig6-A-GAPDH

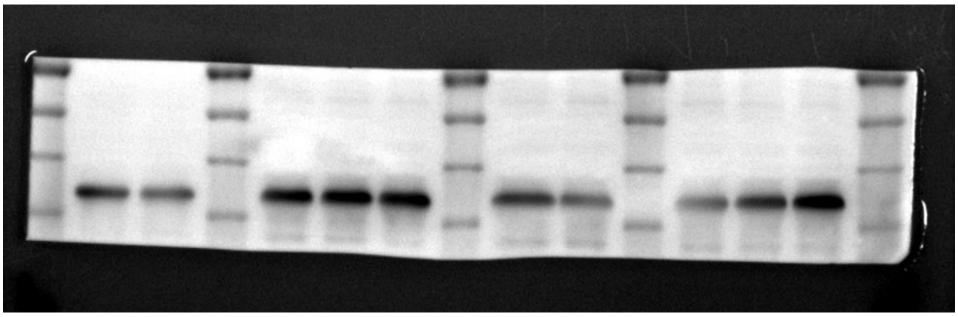

Fig6-N-NAT10

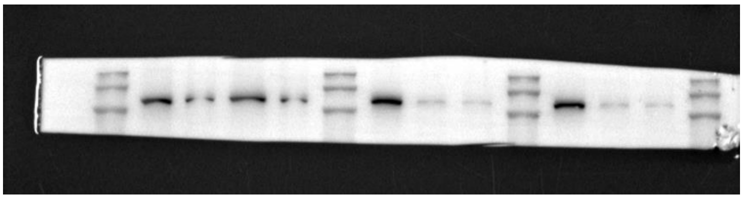

Fig6-N-GLMP

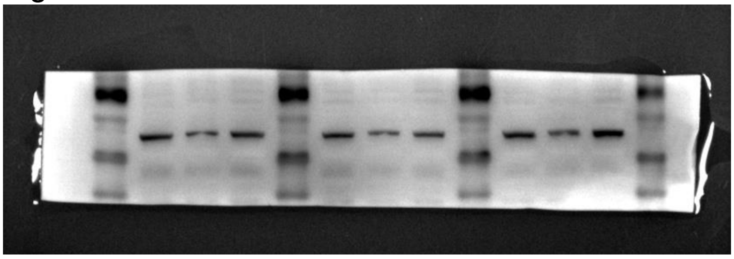

Fig6-N-ERK

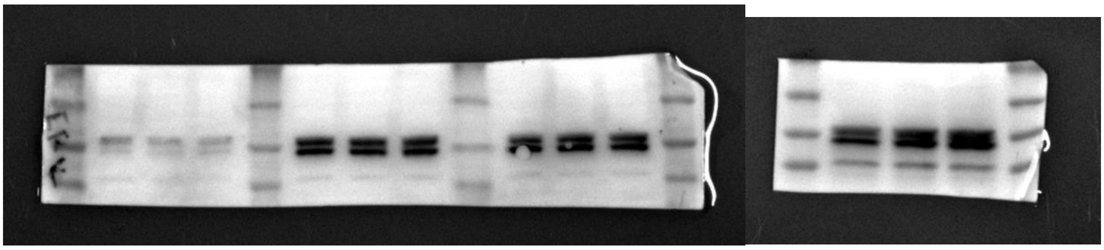

Fig6-N-pERK

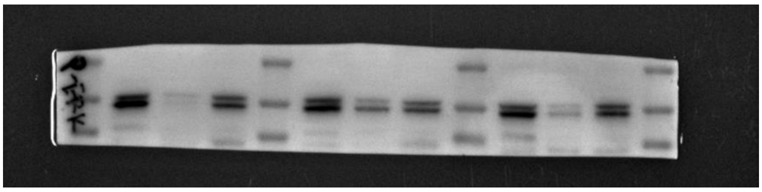

Fig6-N-MEK

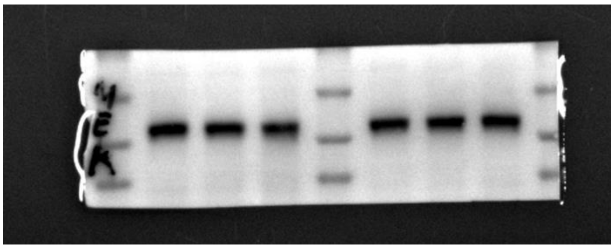

Fig6-N-pMEK

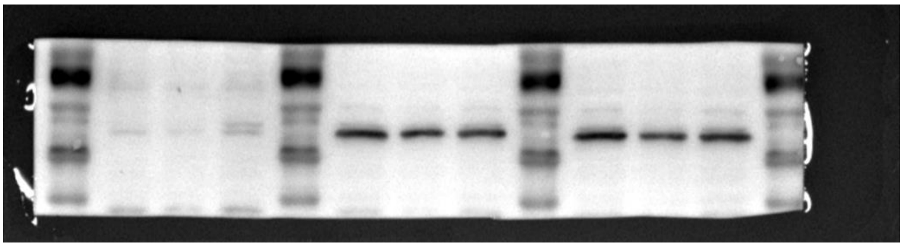

Fig6-N-GAPDH

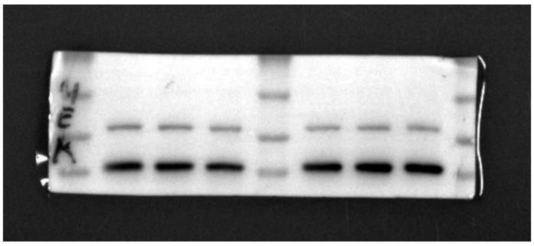

Fig7-D-NAT10

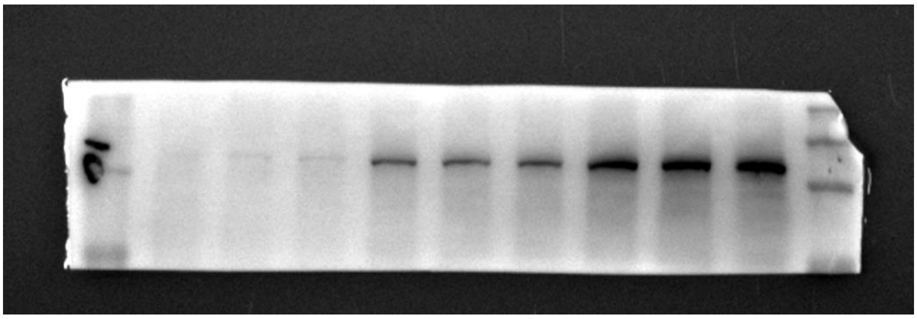

Fig7-D-GLMP

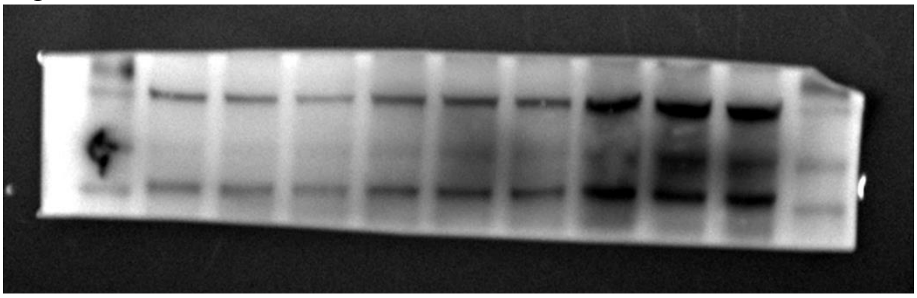

Fig7-D-ERK

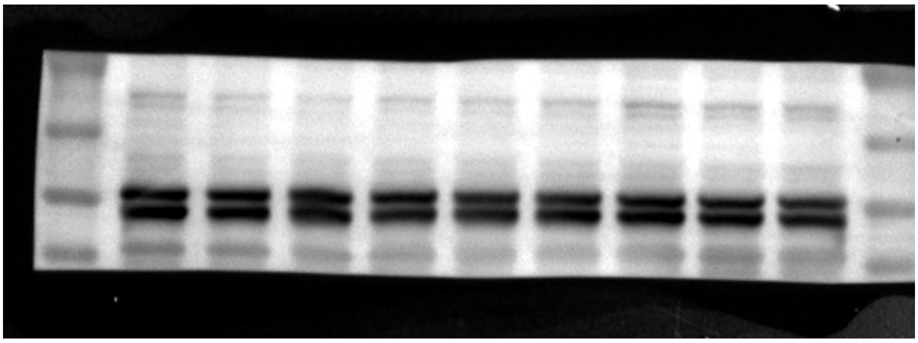

Fig7-D-pERK

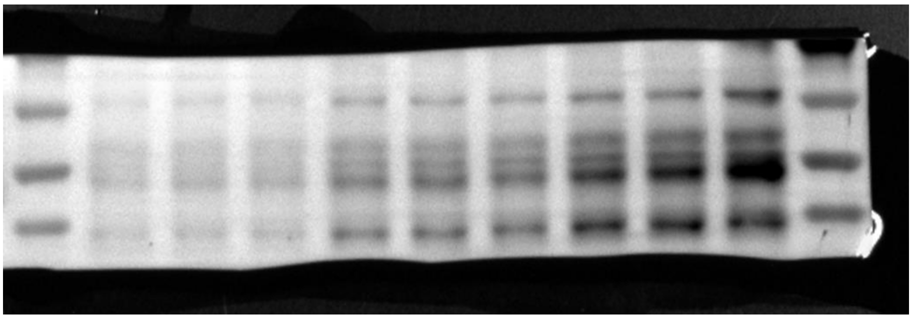

Fig7-D-MEK

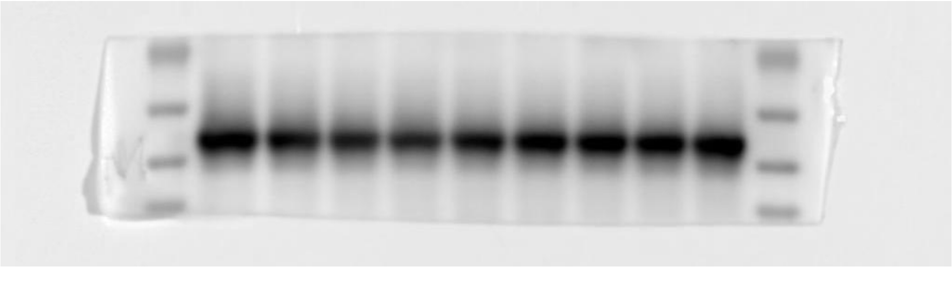

Fig7-D-pMEK

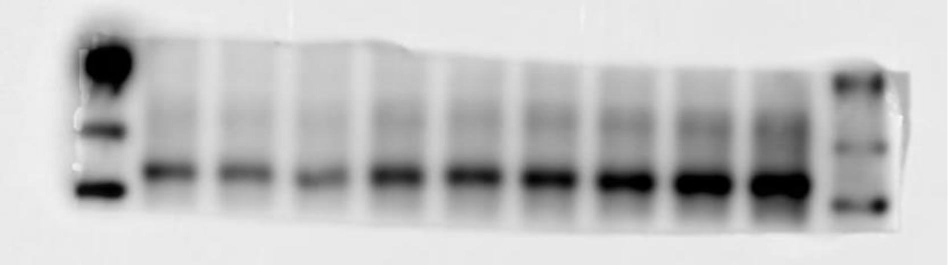

Fig7-D-GAPDH

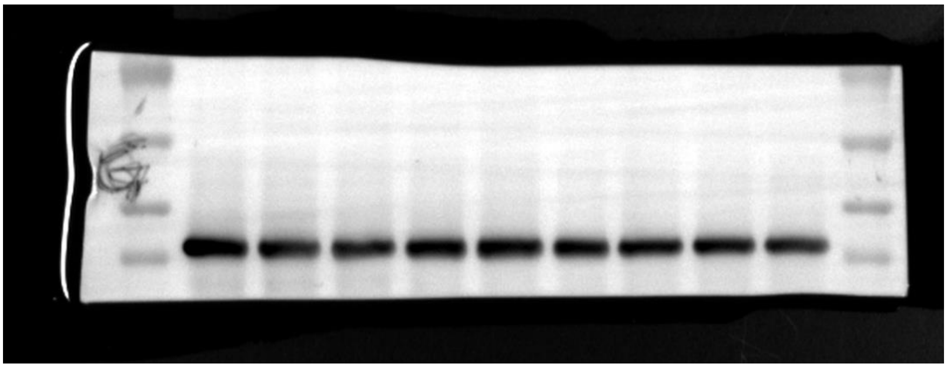

Supplement: Supplementary file 2 — Original Data File [file 41419_2023_6245_MOESM2_ESM.pdf]
